# Cell type specific gene expression analysis of prostate needle biopsies resolves tumor tissue heterogeneity

**DOI:** 10.18632/oncotarget.2744

**Published:** 2014-12-01

**Authors:** Malte Krönig, Max Walter, Vanessa Drendel, Martin Werner, Cordula A. Jilg, Andreas S. Richter, Rolf Backofen, David McGarry, Marie Follo, Wolfgang Schultze-Seemann, Roland Schüle

**Affiliations:** ^1^ Medical Center, University of Freiburg, Department of Urology, 79106 Freiburg, Germany; ^2^ Medical Center, University of Freiburg, Department of Pathology, 79106 Freiburg, Germany; ^3^ University of Freiburg, Department of Computer Science, 79110 Freiburg, Germany; ^4^ Max Planck Institute of Immunbiology and Epigenetics, 79108 Freiburg, Germany; ^5^ Medical Center, University of Freiburg, Department of Medicine I, 79106 Freiburg, Germany

**Keywords:** prostate cancer, RNA detection, living cells, needle biopsy, gene expression, tumor heterogeneity

## Abstract

A lack of cell surface markers for the specific identification, isolation and subsequent analysis of living prostate tumor cells hampers progress in the field. Specific characterization of tumor cells and their microenvironment in a multi-parameter molecular assay could significantly improve prognostic accuracy for the heterogeneous prostate tumor tissue. Novel functionalized gold-nano particles allow fluorescence-based detection of absolute mRNA expression levels in living cells by fluorescent activated flow cytometry (FACS). We use of this technique to separate prostate tumor and benign cells in human prostate needle biopsies based on the expression levels of the tumor marker alpha-methylacyl-CoA racemase (AMACR). We combined RNA and protein detection of living cells by FACS to gate for epithelial cell adhesion molecule (EPCAM) positive tumor and benign cells, EPCAM/CD45 double negative mesenchymal cells and CD45 positive infiltrating lymphocytes. EPCAM positive epithelial cells were further sub-gated into AMACR high and low expressing cells. Two hundred cells from each population and several biopsies from the same patient were analyzed using a multiplexed gene expression profile to generate a cell type resolved profile of the specimen. This technique provides the basis for the clinical evaluation of cell type resolved gene expression profiles as pre-therapeutic prognostic markers for prostate cancer.

## INTRODUCTION

In men, prostate cancer is the most commonly diagnosed cancer and is the second leading cause of cancer related deaths [1]. For 2012, the incidence was estimated to be 238,000 and the mortality to be 29,000 in the USA. Even though 5-year survival rates exceed 98%, appropriate risk adapted therapies remain a challenge in identifying and separating patients with low and high-risk cancer [1]. Patients with slowly progressing tumors with a low risk for incurable metastatic disease have to be separated from quickly progressing tumors with a high risk for incurable metastatic disease. Data from one of the largest PSA screening trials (European Randomized Study of Screening for Prostate Cancer: ERSPC) [2] of more than 180,000 men estimated that rates for over-treatment are up to 50% in low risk groups. Moreover, high mortality rates in the high-risk group clearly demonstrate a need for improved therapeutic strategies.

Current pre-therapeutic risk assessment is based on limited clinical parameters such as histological grading of needle biopsies by the Gleason score, clinical TNM classification and blood serum-based PSA levels [3]. The needle biopsy is used as the gold standard for prostate cancer diagnosis, yet provides limited biological material (ca. 1×1×10mm). The cellular heterogeneity of the tissue consists of the epithelial tumor as well as benign cells, mesenchymal cells (stromal cells, myofibroblasts, endothelial cells, lipocytes) and lymphocytes, which contaminate the bulk tissue and decrease its usefulness for analysis [4]. Several published RNA-seq or microarray data sets have used bulk tumor tissue to generate profiles in order to classify prostate tumors [5, 6]. Often, only a small distinct tissue section of the tumor is selected for the analysis, which might underestimate the heterogeneity of the tumor within the organ [7]. Additionally, the proportion of contaminating cells such as mesenchymal cells in a tumor sample can significantly influence the expression profile of the sample, even if corrected for genes typically expressed in such cells [8]. Micro laser dissection can resolve the cellular heterogeneity and allows for RNA-seq analysis, but is often limited by poor RNA quality due to the fixation process [9]. Furthermore, cells cannot be used for *in vitro* culture assays after fixation.

Until recently, isolation of living cells was primarily limited to detection based on the expression of cell surface proteins. Novel, functionalized gold nano-particles allow for the isolation of living cells based on absolute mRNA expression levels of a specific target [10]. Alpha-methylacyl-CoA racemase (AMACR) is routinely used as a biomarker in prostate cancer diagnosis as it is overexpressed in 80% [11] of prostate cancers at the protein and mRNA level [12, 13]. However, AMACR overexpression is typically also seen in HGPIN (high grade prostatic intraepithelial neoplasia), up to 21% of typical benign glands, in 10–79% of partial atrophy and 10% of adenosis [14]. On the other hand certain prostate cancer subtypes such as foamy gland carcinoma, atrophic and pseudohyperplastic carcinoma show low expression of AMACR [15]. We have to take into account that all the former entities might even coexist within the same specimen. Nonetheless, as such AMACR represents the best studied and routinely used potential target to identify living tumor cells using functionalized gold-nano particles (see methods). This technique might allow to further discriminate between tumor and benign cells, which both express the routinely used EPCAM cell surface protein. Isolated cell populations can now be separately analyzed for gene expression profiles. Advances in the technique for gene expression analysis allow for the detection of gene expression profiles down to the single cell level [16–19]. This allows for analyzing small samples from sparse input material such as needle biopsies.

In this study, we present a technique to characterize a prostate tumor by cell type resolved gene expression profiling from low input material such as needle biopsies. Distinct cell types were isolated simultaneously from needle biopsies. These cells were viable and were either used for in vitro culture or for multiplex gene expression analysis. Multiple biopsies were analyzed to cover different sections of the tumor.

## MATERIAL AND METHODS

### Analysis of RNA-seq data sets

Two independent human prostate cancer RNA-seq studies with cancer and matched benign samples from 10 patients per study were analysed [6, 43]. Both data sets were processed separately as follows: raw sequencing reads were mapped to the human genome (assembly hg19) with TopHat2 with first aligning reads against the transcriptome (Ensembl v65 gene annotation) (further non-default TopHat2 parameter chosen according to study-specific read length and fragment length distributions: “-r 140 —mate-std-dev 20 —segment-length 19” for the former and “-r 150 —mate-std-dev 38 —segment-length 18” for the latter data set). Sequencing reads per annotated gene (Ensembl v65) were counted with htseq-count [44]. Differentially expressed genes between cancer and benign prostate samples were determined with DESeq2, taking into account the patient-wise pairing of tumor and benign sample as additional factor.

### Reverse transcription and pre-amplification

Cells were sorted directly into 5μl 2x reaction mix (CellsDirect one-step qRT-PCR Kit, Life Technologies, cat. 11753-500). Cells were frozen at –80°C for efficient lysis for 2 h. RT/TAQ polymerase, polyT primer and all specific TaqMan assays (Life Technologies) were added (0.2x) for reverse transcription and 22 cycles of pre-amplification (15′ 50°, 2′ 90°C, 15′' 95°C, 4′ 60°C). Pre-amplified samples were diluted 1:5 with DEPC water and stored at −20°C.

### qRT-PCR

For gene expression analysis, 1μl of pre-amplified sample was used for qRT-PCR. Specific TaqMan assays (1x, Life Technologies) and TaqMan Fast Universal RNX 2x were used in 20μl total volume for amplification (2′70°C, 2′ 95°C, 40x: 5′' 96°C, 20′' 60°C).

### Multiplex qRT-PCR (48.48 dynamic array) on Biomark analyzer (Fluidigm)

Preamplified cDNA and TaqMan assays were mixed with appropriate loading buffer and loaded onto a 48.48. dynamic array for gene expression (Fluidigm) according to the manufacturer's instructions. Amplification was performed on a Biomark analyzer (Fluidigm) using a standard protocol (2′ 50°C thermal mixing, 10′ 95°C denaturation, 40x: 15′' 95°C, 1′ 60°C).

### Hierarchical clustering

Results from multiplex analysis on 48.48 dynamic array were exported as heatmaps and analyzed using the SINGuLAR^TM^ script (Fluidigm) for R. A detection limit was defined and CT values were converted into (Detection limit – CTvalue) for easier visualization. Negative values are represented as zero. Euclidean hierarchical clustering was performed. Values range from 0 to 12 with increasing expression level and are color-coded from red to white.

### Normalization

In the standard qRT-PCR experiments, expression values were normalized to AMACR low expressing populations. Considerable variation in the stochastic expression levels of housekeeping genes - and all other genes - at the single cell level makes the use of housekeeping genes not feasible (Fig. S3). We expected that the stochastic variation in expression levels would also affect cell numbers such as the 200 cells used in this study. The precise control of the number of input cells by FACS ensures the same amount of input RNA for the analysis.

### Needle biopsy processing

Written consent was obtained from patients prior to any treatment including radical prostatectomy. The work was approved by a local ethics committee. Radical prostatectomy specimens were received and processed within 10 minutes after the dissection by a surgical-pathologist. Specimens were incised dorso-ventrally and needle biopsies were retrieved by conventional punch technique. Histological validation using H&E staining was performed on cross sections from the area where needle biopsies were retrieved. Needle cores were cut into 1×1mm pieces and incubated with 1:10 Collagenase (10mg/ml) in DMEM supplemented medium overnight at 37°C. Cores were washed and incubated with 5% Trypsin EDTA for 10 minutes at 37°C. Cores were separated into single cells by mechanical force (18 and 20G needles) and filtration (40μm). Cells were resuspended in 1ml DMEM and either used for analysis or frozen in liquid nitrogen.

### Culturing of primary cells

Primary epithelial cells were sorted directly into PREGM medium (Lonza) supplemented with 15% Matrigel^TM^ (BD Biosciences). 500 ml PREGM Medium includes the following factors: BPE, 2 ml; Hydrocortisone, 0.5 ml; hEGF, 0.5 ml; Epinephrine, 0.5 ml; Transferrin, 0.5 ml; Insulin, 0.5 ml; Retinoic Acid, 0.5 ml; Triiodothyronine, 0.5 ml; GA-1000, 0.5 ml. Matrigel is a commercially available extracellular matrix analoga. 15% matrigel provided highest highest culturing success.

### SmartFlares (SF; MerckMillipore)

In brief, a 27 bp complementary target sequence is conjugated to a gold nanoparticle. A shorter complementary reporter strand binds to the target strand and is conjugated with a fluorophore (Cy5). The fluorophore is quenched by the gold nanoparticle when in the bound state. An endogenous target sequence with a higher binding affinity (due to its increased length) will replace the reporter strand and lead to the emission of a fluorescent signal, which can be detected by conventional FACS. No toxic or immunologic reactions induced by the gold nanoparticles have been reported to date [10]. The endo- and exocytosis machinery of living cells facilitates cellular uptake and release of the gold nanoparticles. This feature allows for cultivation of the cells after FACS isolation.

Cells were incubated with 1:20 diluted (PBS) SmartFlare (AMACR-Cy5 (SF985) or Scramble-Cy5 (SF102)) for 6 h in 1ml DMEM at 37°C (light protected).

### Fluorescent activated cell sorting (FACS)

Cell sorting was conducted with a FACSAria III cell sorter (BD Biosciences).

### Antibodies and dyes

We used the following antibodies:

Anti-Human CD326 (Ep-CAM), Brilliant Violet 421, clone 9C4 (Biolegend, cat. 324219), Anti-Human CD45, PerCP-Cy5.5, clone HI30 (eBiosciences, cat. 45-0459)

YOPRO-1 iodide (Life Technologies, cat. Y3603), Anti-Human p63 / P504S, Klon: BC4A4, polyclonal (DCS diagnostics)

## RESULTS

Two independent RNA-seq data sets [6, 43] from human prostatectomy samples were analyzed. Only samples where tumor and benign control tissue matched to the same patient were included (*n* = 20). A total of 322 genes were identified as significantly differentially expressed in both data sets at a false discovery rate (FDR) of 0.1. The fold change in both data sets is highly correlated (Spearman's correlation coefficient ρ = 0.9037) (Fig. S1). A spearman coefficient of 0.9037 indicates a variation in the expression levels, which is most likely attributed to the biological variation of the two different sample populations (Fig. S1). Finally, 36 / 322 genes were selected according to a mean gene expression > 300 normalized read counts, log2 fold change > 1 for up-regulated and < −1 for downregulated genes (Prenser et al. data set [6]), and known regulatory or marker functions in prostate cancer. Additionally, marker genes for epithelial to mesenchymal transition (EMT), stemness and cell lineage for epithelial, stromal and lymphatic cells were included. Stemness and EMT genes are involved in metastatic and drug resistance pathways in various cancer entities [45]. Taqman primers for the selected genes were purchased from Life Technologies. Rigorous selection (efficiency between 90–110%) left 33 genes available for further analysis (see methods for complete list).

**Figure 1:**
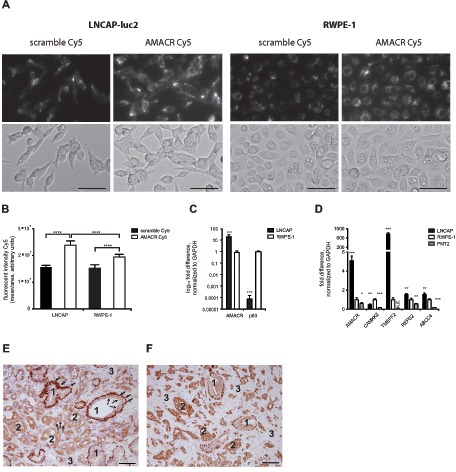
**(A)** A total of 5000 cells per well were incubated with AMACR-Cy5 and Scramble-Cy5 SmartFlare and tracked for 28 h by time-lapse microscopy (upper panel: Cy5, lower panel: bright field, 28 h); bar = 100μm; **(B)** Cy5 fluorescence intensity is significantly higher at 24 h in LNCAP cells compared to RWPE-1 cells (*p* < 0.0005, 8 replicates) and Scramble in both cell lines (*p* < 0.0005); **(C–D)** Selected tumor markers are significantly expressed in tumor cells (LNCAP) compared to benign cells (RWPE-1 and PNT2), p-values are indicated by stars: * = *p* < 0.05, **** = *p* < 0.00005) **(E)** typical heterogeneous cellular histology of prostate cancer tissue (immunohistological staining with AMACR and p63): 1 = benign gland, 2 = tumor gland, 3 = mesenchymal cells, arrows = p63 positive basal cells, dotted arrow = AMACR positive luminal cells (magnification 20x; bar = 100μm).

AMACR was among the 5 most up-regulated genes of the 322 genes identified and therefore was selected for customized SmartFlare design. The target sequence was directed against nucleotide 97–123 (according to the manufacturer). In a preceding experiment we confirmed the intracellular uptake of AMACR SmartFlare and a scramble control using confocal microspcopy in LNCAP cells after 4 h of incubation (Fig. S2). The intracellular AMACR signal correlates with FACS analysis, where 91% of the cells stained positive. To test the specificity of our AMACR SmartFlare oligos in cell culture systems, we selected two cell lines with significantly different expression levels of AMACR: the prostate tumor cell line LNCAP (high AMACR expression) and the benign prostate cell line RWPE-1 (low AMACR expression). qRT-PCR analysis resulted in an approximately 40-fold difference in AMACR expression between LNCAP and RWPE-1 cells when normalized against GAPDH. SmartFlares detect only absolute levels of mRNA expression and hence normalising qPCR data relative to a housekeeping gene can be misleading unless the input number of cells is precisely controlled. Therefore, we performed a cell culture-based *in vivo* validation of the AMACR SmartFlare specificity. LNCAP and RWPE-1 cells were incubated with 20μl of 1:20 diluted AMACR or a scrambled SmartFlare oligo as a control and monitored over 24 h for the absolute fluorescent intensity signal per cell using fluorescent time-lapse microscopy. Cells were automatically tracked by additional nuclear staining with Hoechst dye. Figure 1A demonstrates significantly higher fluorescence intensity after 24 h in LNCAP cells compared to RWPE-1 cells (*p* < 0.0005). The scramble signal is detectable at comparable levels in both cell lines and significantly lower compared to the AMACR signal (*p* < 0.005).

Next, we validated the expression levels of AMACR, CAMKK2, TMEFF2, REPS2 and ABCC4 identified in the above-mentioned RNA-seq data in the tumor cell line LNCAP and two benign cell lines RWPE-1 and PNT2 cells. All five tested genes showed significant up-regulation in LNCAP cells compared to RWPE-1 or PNT1A cells (*p* < 0.05). We also showed that the AMACR and p63 protein staining pattern in prostate tumors and benign glands mirrors the mRNA expression pattern in LNCAP cells and RWPE-1 cells. Tumor glands lack a basal cell layer and therefore do not stain positive for p63. In contrast, luminal benign epithelial cells show weak or absent staining for AMACR, whereas epithelial tumor cells show in most cases strong staining for AMACR instead (Fig. 1E and 1F). However, when AMACR staining intensity is compared between the two representative samples demonstrated in Fig. 1E and 1F it becomes obvious that the difference between benign and tumor cells is less prominent in E compared to F. This again underlines the heterogeneity in the AMACR expression. The gene expression pattern (up-regulated AMACR mRNA and down-regulated p63 mRNA) in LNCAP cells (normalized to benign RWPE-1 cells) represents the protein staining pattern (up-regulated AMACR and absent p63 protein) in primary prostate cancer tissue with high AMACR expression.

**Figure 2:**
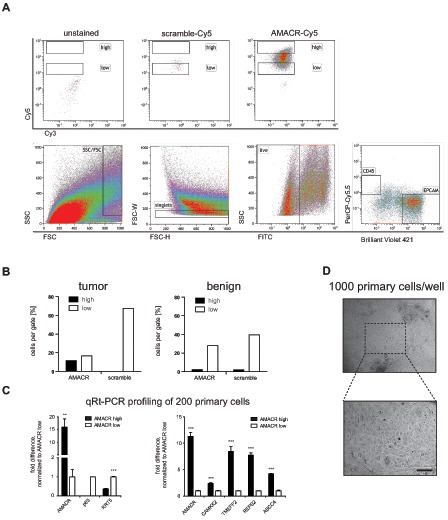
AMACR detection using SmartFlares in human prostate cancer needle biopsy **(A)** upper panel: Specific AMACR-Cy5 signal in EPCAM positive cells (unstained and scramble-Cy5 controls); lower panel: gating strategy for size exclusion, singlets, living cells and EPCAM positive cells; **(B)** Enrichment of AMACR-CY5 positive cells in *high* gate compared to scramble-Cy5 and benign tissue; **(C)** AMACR-high expressing cells express significant levels of tumor markers and decreased levels of basal cell markers p63 and cytokeratin 5 (KRT5); **(D)** 1000 epithelial cells were expanded for several days with typical morphology (upper panel: 10x, lower panel 20x) in PREGM supplemented with 15% Matrigel; bar = 100μm.

Next, single cell suspensions from fresh needle biopsies from human prostatectomy specimens were stained with AMACR SmartFlares and fluorescence-labeled antibodies against CD45 and Epcam. Single living cells were gated for CD45 positive lymphocytes and Epcam positive epithelial cells. CD45/Epcam double negative cells represent mesenchymal cells. Epcam positive cells were further gated into AMACR high and low expressing cells (Fig. 2A). We observed significant enrichment of AMACR positive cells in the AMACR high compared to scramble control (Fig. 2B). We did not observe any enrichment in a benign control sample isolated from the same patient (Fig. 2B). Next, 200 cells from the high and low gate were sorted for qPCR analysis for a subset of tumor markers such as AMACR, CAMKK2, TMEFF2, REPS2 and ABCC4. Single tube assays were used for reverse transcription and pre-amplification. We observed higher expression of the tumor markers in the AMACR high expressing cells, which were identified by AMACR SmartFlares. Additionally, AMACR high expressing cells showed no expression of the basal cell marker p63 and higher expression of the luminal cell marker cytokeratin 8 (KRT8). This expression pattern is typical for prostate tumor cells and is in agreement with previous reports [46]. Additional cells were sorted, cultivated at a density of 1000 cells per 96-well and expanded for several days (Fig. 2D). Cells were cultivated in commercially available epithelial stem cell medium (PREGM, Lonza) supplemented with 15% matrigel (for detailed information see methods). Epcam positive epithelial cells, which comprise AMACR high and low expressing cells, represent only 17, 30% of all living cells (Table S4).

**Figure 3:**
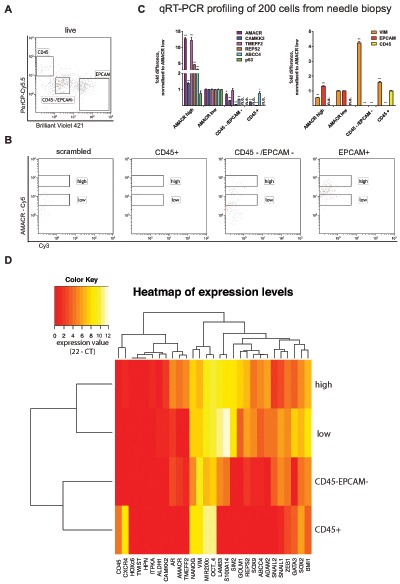
Cell type resolved analysis of human prostate cancer needle biopsy **(A)** Gating strategy for CD45 positive lymphocytes, Epcam positive epithelial cells and CD45/Epcam double negative stromal cells; **(B)** AMACR-Cy5 signal detected in indicated subpopulations (CD45+, CD45–/EPCAM–, EPCAM+ and scramble); **(C)** Gene expression analysis for tumor marker shows significant expression in AMACR high cells compared to AMACR low cells (normalization to AMACR low cells, right panel). Gene expression analysis for lineage markers (Vimentin = stromal cells, CD45 0 lymphocytes, Epcam = epithelial cells) correlates to protein expression-based FACS separation; **(D)** hierarchical euclidean clustering from gene expression analysis of 29 genes in each population separates the sorted four populations (expression value = 22 – CT).

We decided to build upon the existing model and further characterize the cellular populations isolated from our samples. A separate sample was prepared under the same conditions except that four populations were sorted in parallel using a 4-way sort algorithm to minimize loss of cells. A total of 200 cells each from CD45 positive lymphocytes, CD45/Epcam double negative mesenchymal cells, Epcam positive AMACR high cells and Epcam positive AMACR low cells were sorted. We used the same tumor markers as before and included additional lineage markers such as Vimentin (stromal cells), CD45 (lymphocytes) and Epcam (epithelial cells). We confirmed that the sorted cell populations were identified by the expression of specific marker genes (Fig. 3C). Expression levels were normalized to AMACR low cells. In line with our previous observations (Fig. 2), the tumor markers AMACR, CAMKK2, TMEFF2, REPS2 and ABCC4 were significantly expressed in AMACR high cells as compared to AMACR low cells. Some tumor markers such as AMACR, TMEFF2 and ABCC4 were also expressed in mesenchymal cells or lymphocytes at levels comparable to AMACR low cells. The analysis of the AMACR-Cy5 fluorescence intensity in the gates for CD45 and Epcam/CD45 double negative cells were selected to represent the expression levels observed by qPCR (Fig. 3B). An additional 200 cells from the same needle biopsy were sorted and analyzed for the expression of 29 genes (Methods Table 1 except with additional lineage marker). Gene sets representative for stemness and epithelial to mesenchymal transition (EMT) were included, which provide information on the metastatic potential of the tumor cells. Also, genes that are commonly found to be down-regulated in prostate cancer were included (Methods Table 1). A hierarchical clustering analysis separated the four populations (Fig. 3D) resembling the qPCR results presented in Fig. 3C.

**Table 1:** Gene targets

Gene	TAQMAN Assay ID (Life Technologies)
**Tumor marker**	
**upregulated**	
ADAM2 [20]	HS00155182_m1
AMACR [6]	Hs01091292_m1
TMEFF2 [21]	Hs01086906_m1
HPN [22]	Hs01056332_m1
HOXC6 [23]	Hs00171690_m1
CAMKK2 [24]	Hs00198032_m1
ABCC4 [25]	Hs00988717_m1
REPS2 [26]	Hs00190932_m1
**downregulated**	
LAMB3 [27]	Hs00165078_m1
S100A14 [28]	Hs04189107_g1
ITPKA [29]	Hs00176658_m1
GATA3 [30]	Hs00231122_m1
**Epithelial Mesenchymal Transition (EMT) marker**	
**upregulated**	
ZEB1 [31]	Hs00232783_m1
SNAIL1 [32]	Hs00195591_m1
SNAIL2 [32]	Hs00950344_m1
TWIST [32]	Hs00361186_m1
MIR200c [32]	Hs04231534_s1
**Stemness marker**	
**upregulated**	
SOX2 [33]	Hs01053049_s1
SOX9 [34]	Hs01001343_g1
ALDH1 [35]	Hs00946916_m1
BMI1 [36]	Hs00995536_m1
OCT-4 [37]	Hs00999634_gH
NANOG [37]	Hs02387400_g1
**Lineage marker**	
**upregulated**	
Vimentin [38]	Hs00185584_m1
CXCR4 [39]	Hs00237052_m1
CD45 [40]	Hs04189704_m1
AR [41]	Hs00171172_m1
**Additional Lineage marker**	
**upregulated**	
Epcam	Hs00901885_m1
KRT5 [42]	Hs00361185_m1
KRT8 [42]	Hs01595539_g1
P63 [42]	Hs00978343_m1

**Figure 4:**
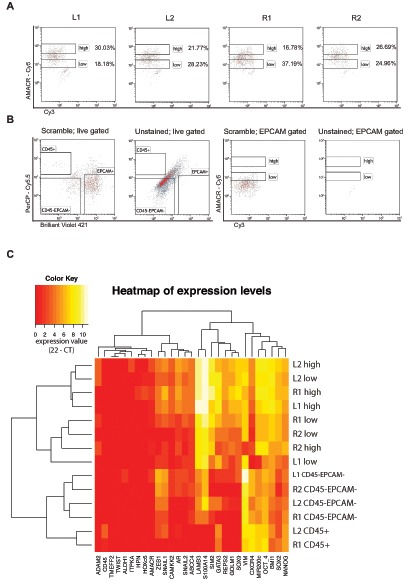
Cell type resolved analysis of four human prostate cancer needle biopsies (L1, L2, R1, R2; L = left side, R = right side of the specimen) **(A)** Proportions of cells in AMACR *high* or *low* gates vary in the analyzed needle cores; **(B)** Scramble and unstained controls in the live gate confirm the presence of lymphocytes, stromal cells and epithelial cells (left two plots), while unstained and scrambled controls in the Epcam gate (right two plots) confirm the specificity of the signal detected in A), **(C)** hierarchical euclidean clustering from gene expression analysis of 29 genes in each population generates clusters for lymphocytes, stromal cells and epithelial cells from all biopsies, lymphocytes were only present in R1 and L2 (expression value = 22 – CT).

To account for the multifocal occurrence of prostate cancer, four separate needle biopsies (L1, L2, R1, R2) from another prostatectomy specimen were analyzed using the same setup as before (Fig. 4B). Distribution of cells in the AMACR high and low gates did show some variation in the four needle biopsies and only two biopsy cores contained detectable CD45 positive lymphocytes. Both findings can be explained by the expected heterogeneous cellular composition in each biopsy (Fig. 4A). Expression levels were analyzed by hierarchical clustering, and clearly separated mesenchymal cells and lymphocytes from epithelial cells (Fig. 4C). Epithelial cells were also clustered in AMACR high and low expressing cells. We could also detect the already described heterogeneous expression pattern of AMACR on the gene expression level. From biopsy L2 the AMACR low expressing cell population did show the gene expression signature from tumor cells. Vice versa the AMACR high expressing cell population from biopsy R2 did show a benign expression profile (Fig. 4C). These findings further support the reported cellular heterogeneity within separate biopsies represented by different proportions of cell types per biopsy. Distribution of cell types within the needle biopsies are described in Table S4.

## DISCUSSION

We can conclude that AMACR mRNA expression level-based separation of epithelial cells generates cell populations that closely resemble tumor and benign prostate cells, with respect to their gene expression profile. Even representing the expected heterogeneity of AMACR expression within the same specimen.

It is the first time that this technology has been applied to primary prostate tissue and small input material such as a needle biopsy. We selected AMACR as a target in this study because of its routine use in cancer diagnostics and the good correlation of expression at the mRNA and protein level [12, 13]. However, this technology can be applied to any target of interest. The increasing number of individual primary prostate tissue transcriptomes from RNA-seq provides the basis for the selection of such targets. Unfortunately, the majority of the above data is acquired from bulk tissue analysis and may not represent the expression levels found in a cell type resolved analysis. For the majority of samples analyzed, we observed only one population of AMACR positive cells, which was stretched over more than one log_10_ step of fluorescence intensity. These findings most likely represent a continuous regulation of AMACR expression within the epithelial cell population. Technical explanations such as hydrolysis of the SmartFlare reporter oligo or detection of splice variants could account for such a signal distribution. Unspecific signals induced by hydrolysis are controlled by scramble SmartFlares, which showed significantly lower fluorescent signals than those detected in the AMACR high gate. The AMACR SmartFlare was designed to detect exon one, which is included in all known splice variants. No single cell analysis data regarding AMACR expression in living human prostate tumor cells are available for comparison.

A limitation of the expression profiles of the separated populations occurs when attempting to correlate the data with detailed histological analysis. The cross section of the prostatectomy sample showed a heterogeneous tumor tissue with tumor and benign glands and proportions of mesenchymal cells and inflammatory cells such lymphocytes. However, no exact estimation can be made to analyze the cellular composition along the needle biopsy. For future studies, improved biopsy technologies are needed, which would allow for simultaneous analysis of living cells and histological validation. We currently explore the use of a needle punch system to generate two tissue cylinders in very close proximity (< 1mm). One cylinder can be used for the analysis of living cells and the other one for standard histological analysis. A second method we currently explore is to cut one tissue biopsy cylinder longitudinally and analyze the two halfs separately. The latter methods would then even further reduce the input material. However we could already recover sufficient number of cells and paraffin embedded material for downstream analysis in a feasibility experiment (data not shown).

The use of 200 cells from each population allows for the detection of a large number of transcripts and samples in a cost efficient way. As yet, this does not allow resolution at the single cell level. The number of detectable genes per cell decreases at the single cell level, which is mainly attributed to highly stochastic expression of certain transcripts in a single cell at a given time point [16, 17]. From 48 selected genes for single cell expression analysis in 23 LNCAP cells, only 16 were expressed in at least 10% of the cells (data not shown), an observation that has been published previously [17]. By using 200 cells, we can account for the stochastic single cell effect and allow for the detection of a broader gene expression profile in a defined cell population. For certain low abundant subpopulations such as tumor stem cells or infiltrating lymphocytes, single cell analysis remains a valuable tool that can be applied using our method [17–19].

The multiplex gene expression analysis on a 48.48 dynamic array is limited to 48 genes and 48 samples. However, it represents a cost efficient technology for analyzing a large number of samples with minimal reagent input and high precision due to the microfluidic driven assembly of the nano liter scaled reaction volumes. To further increase the power of this system in order to reveal novel interaction networks, potentially to be used as prognostic criteria, RNA-seq technology has to be applied via adopted single cell protocols [19, 47].

Cell populations referred to as mesenchymal cells in this work are known to consist of further subpopulations such as stromal cells, endothelial cells, myofibroblast cells and cancer associated fibroblasts (CAFs) [48]. The same holds true for CD45 positive lymphocytes, which can be further subdivided into CD3+, CD4+, CD8+ and FoxP3+ cells [49]. These subpopulations will have to be taken into account in future studies.

Using a cell type resolved analytical method of living cells to dissect prostate tumor tissue can overcome several previous limitations and improve the quality of tissue analysis post-biopsy: Living cells from primary tissue can be analyzed for a gene expression signature and, from the same population of cells, could be used for functional assays such as drug screening, murine xenografts or three-dimensional tissue cultures to examine drug response within the complex interaction of different cell types. Such analyses cannot be performed with fixed tissue biopsies. Further more, potential “omics” analyses from living cells provides a higher quality of input material compared to formalin or alcohol fixed tissue.

A commercially available gene expression signature assay for prostate cancer has been recently validated in several patient cohorts and showed superior prediction for prostate cancer disease recurrence after the initial treatment when compared to clinical and histological variables [50]. However, it has focused on a single tumor focus per patient, which has been analyzed as a bulk sample from formalin fixed tissue. Our method has the potential to even further refine the prediction accuracy of such a test assay by dissecting and analyzing the heterogeneity of different cell types at multiple sites within the tumor tissue. Different cells types can be analyzed separately at multiple sites of the tumor. Furthermore, different gene expression signatures from distinct cell types such as tumor cells, benign cells, mesenchymal cells and lymphocytes can be correlated to identify better prognostic and therapeutic strategies. A limitation of using living cells is that data has to be collected prospectively with follow up periods of five to ten years to correlate gene expression signatures to clinical outcomes such as disease recurrence, treatment response or death from cancer. Still, several important issues for clinical decision making such as the presence of metastatic disease at the time of diagnosis, the identification of the biopsy of origin of the metastases or the initial treatment response, could be answered with significantly shorter follow up.

In summary, an integrated cell type resolved gene expression analysis derived from multiple histological validated needle biopsies from the same organ provides the basis to further analyze novel interaction networks, define prognostic signatures and identify therapeutic targets in heterogeneous tumor tissue. Such tools will help to identify prostate cancer patients at high risk for tumor progression or death from cancer and treat them effectively as well as to prevent unnecessary treatment of patients with indolent prostate cancer.

## SUPPLEMENTARY FIGURES AND TABLE


