# Supplementary material for: Cell type specific gene expression analysis of prostate needle biopsies resolves tumor tissue heterogeneity
Source: Oncotarget. 2014 Dec 1;6(2):1302–14. doi: 10.18632/oncotarget.2744 (PMC4359234; doi:10.18632/oncotarget.2744)
Supplement: Supplementary file 1 [file oncotarget-06-1302-s001.pdf]

SUPPLEMENTARY FIGURES AND TABLE

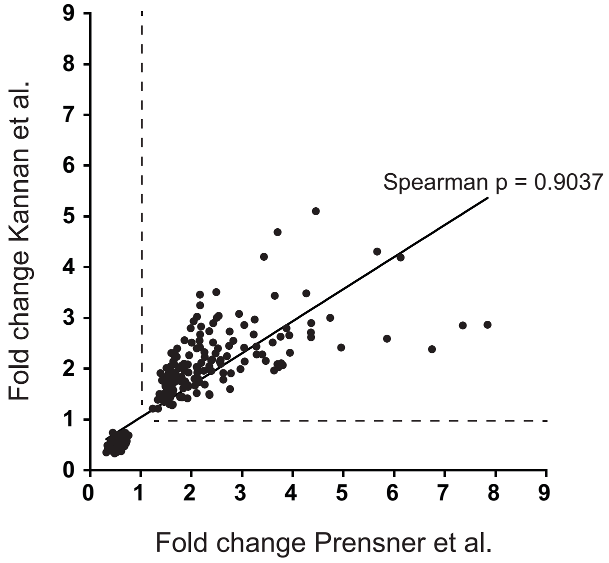

Supplementary Figure S1: Fold changes of the 322 significantly differentially expressed genes at a FDR of 0.1 from the indicated RNA-seq data sets. The change in gene expression strongly correlates (Spearman's correlation coefficient ρ = 0.9037).

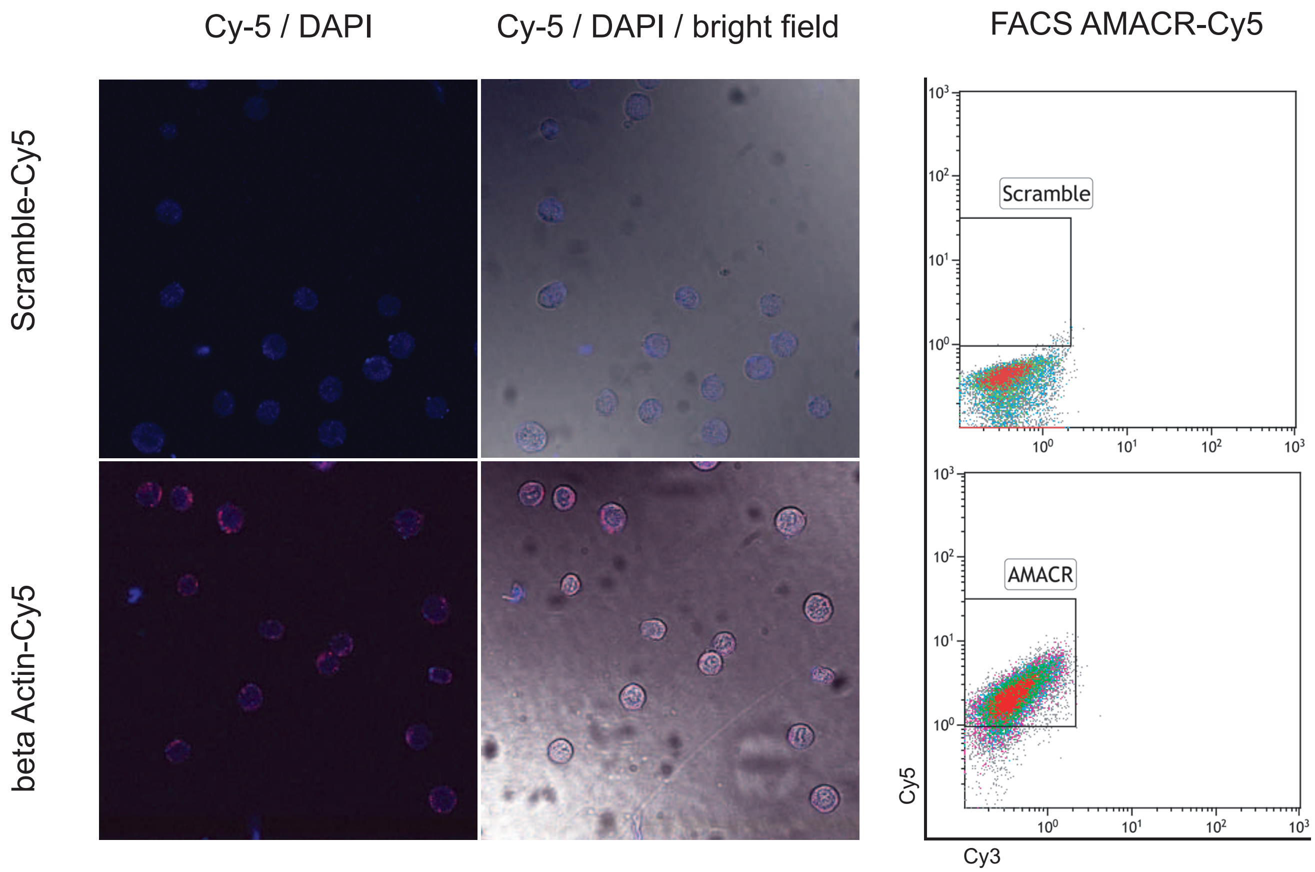

Supplementary Figure S2: Intracellular uptake of beta Actin SmartFlare in LNCAP cells. Upper panel: scramble-Cy5 control, lower panel: beta Actin-Cy5, left panel: Cy5/ DAPI overlay, right panel: Cy5/DAPI and bright field overlay. Signal is only detected within the cellular borders determined in bright field; confocal microscopy, bar = 50µm.

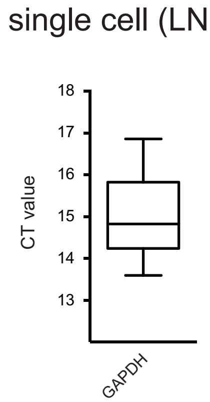

Supplementary Figure S3: Single cell gene expression analysis for GAPDH of 23 LNCAP cells shows strong variation between cells.

Supplementary Table S4: Cellular distribution of CD45 positive lymphocytes, CD45/Epcam- Stromal cells and Epcam + epithelial cells in examined needle biopsies. Cell types were gated from living cells. AMACR high and low expressing cells were gated from epithelial cells. Cell type "other" did not show conclusive marker expression of either of the above groups.

Cell type distribution in biopsy samples

**Figure 3**	**% of living cells**			
	**Biopsy**			
Lymphocytes	9,0			
Stromal cells	30,45			
Epithelial cells	17,30			
Other	43,25			
	**% of epithelial cells**			
AMACR high	22,34			
AMACR low	21,28			
**Figure 4**	**% of living cells**			
	**Biopsy R1**	**Biopsy R2**	**Biopsy L1**	**Biopsy L2**
Lymphocytes	1,08	0,0	0,0	2,48
Stromal cells	17,63	14,79	14,36	12,16
Epithelial cells	56,14	53,33	53,46	55,15
Other	25,15	31,88	32,18	30,21
	**% of epithelial cells**			
AMACR high	16,78	26,69	30,03	21,77
AMACR low	37,19	24,96	18,18	28,23
